# Oncologic outcome of multimodality treatment for sinonasal malignancies: An 18-year experience

**DOI:** 10.3389/fonc.2022.958142

**Published:** 2022-09-05

**Authors:** Meng-Yu Chen, Xin Wen, Yi Wei, Lin Chen, Zi-Xuan Huang, Tong Lu, Nian-Zhen Zheng, Jian Li, Wei-Ping Wen, Yi-Hui Wen

**Affiliations:** ^1^ Department of Otolaryngology, the First Affiliated Hospital of Sun Yat-sen University, Guangzhou Key Laboratory of Otorhinolaryngology, Otorhinolaryngology Institute of Sun Yat-sen University, Guangzhou, China; ^2^ Department of Otolaryngology, the Sixth Affiliated Hospital of Sun Yat-sen University, Guangzhou, China

**Keywords:** Sinonasal malignancies, endoscopic surgery, anterior skull base, paranasal sinus, surgical margin

## Abstract

**Purpose:**

The aim of this study was to retrospectively evaluate the oncologic outcomes of sinonasal malignancies (SNMs) of various histologic subtypes and investigate the impact of multimodality treatment on prognosis of SNM.

**Methods:**

SNM patients treated with curative-intent surgery from 2000 to 2018 were included. The primary outcomes were overall survival (OS). Survival was then assessed through Cox proportional hazards models.

**Results:**

Three hundred and three patients were eligible for the analysis. The 5-year OS and event-free survival (EFS) were 61.0% (95% CI: 55.4%–67.1%) and 46.2% (95% CI: 40.4%–52.7%). The 5-year OS was the worst for malignant melanoma and the best for adenocarcinoma. Patients who received surgery had better OS than those who only received radiotherapy and/or chemotherapy. Endoscopic surgery had better OS than the open approach (*p* < 0.05). Microscopically margin-negative resection (R0 resection) significantly benefited OS and EFS (*p* < 0.001). No significant difference in OS was observed between patients who received macroscopic complete resection (R1 resection) followed by adjuvant therapy and patients who received R0 resection. Older age (HR = 1.02, *p* = 0.02), R1 resection (HR = 1.99, *p* = 0.02), sinonasal surgical history of more than 3 months before diagnosis (HR = 2.77, *p* = 0.007), and radiotherapy history (HR = 3, *p* = 0.006) are risk factors for worse EFS.

**Conclusions:**

Curative-intent surgery is irreplaceable in the treatment of SNM. The endoscopic approach is an effective alternative to the open approach. EFS is worse among patients with older age, R1 resection, sinonasal surgical history of more than 3 months before diagnosis, and radiotherapy history.

## Introduction

Sinonasal malignancies (SNMs) are highly heterogeneous malignant tumors derived from the epithelium and mesenchymal cells of the nasal cavity and paranasal sinuses, the pathology of which varies. SNMs are rare tumors constituting 3%–5% of head and neck cancer (1). It has been estimated that the incidence of sinonasal cancers is approximately 0.83 per 100,000 in the United States (2). Unlike other malignancies, there does not appear to be any predominant risk factor in SNM (3). Patients are usually diagnosed at an advanced stage, thus resulting in poor prognosis.

Treatment for SNM is based on multimodality therapy, primarily surgical excision and post-operative radiotherapy as well as chemotherapy (4). Endoscopic sinus surgery plays a significant role in the multidiscipline treatment of SNM. With shorter hospital stays and the avoidance of facial incisions, the application of the endoscopic approach becomes more preferable. However, the complex anatomy of nasal cavity and paranasal sinus makes microscopically margin-negative resection (R0 resection) surgically challenging. Even in traditional open approaches, the margins of surgical resection are positive in 31.6% of patients (5). Therefore, the oncologic surgery principle, including en bloc resection and R0 resection, is not always followed when performing transnasal endoscopic skull base surgery. Some studies even suggested that an en bloc resection was not superior to a piecemeal resection (6, 7). Whether patients could benefit from adjuvant therapy after R0 or R1 resection remains controversial. Therefore, high level of evidence is required to reach consensus in the field of transnasal endoscopic skull base surgery.

This is a series of 303 SNM patients treated in a single tertiary medical institute during an 18-year period. In our study, we aimed to retrospectively compare the oncologic outcomes of SNM patients of various histologic types and investigate the prognosis of SNM patients treated by multimodality therapy with special emphasis on the endoscopic surgical approach.

## Materials and methods

After approval by the institutional review board and registration at the Chinese Clinical Trial Registry (ChiCTR2100048214), a retrospective search of the medical record databases at the Department of Otorhinolaryngology was performed. All patients with biopsy-proven SNMs who underwent curative-intended multimodality treatment between 2000 and 2018 at the First Affiliated Hospital of Sun Yat-sen University were included in this study. Patients who did not complete any follow-up examination, and who were diagnosed as nasopharyngeal carcinoma or lymphoma, such as NK/T cell lymphoma were excluded from our study. **Figure S1** shows the eligibility results. Patients’ medical records were reviewed for information regarding demographics; disease characteristics, such as disease stage; histologic type and grading; and treatment details, including adjuvant therapy, neo-adjuvant therapy, surgical details, and oncologic outcomes, such as disease control, recurrence, and survival. Disease stage was reported using the American Joint Committee on Cancer AJCC Staging Manual, eighth edition (8).

Descriptive statistics for scaled values and frequencies of study patients within the categories for each of the parameters of interest were enumerated with the assistance of commercial statistical software. We calculated overall survival (OS) from the time of presentation to the date of either last contact or death. Patients who were alive at last contact or lost to follow-up were censored. Event-free survival (EFS) was calculated from the date of presentation to the date of recurrence of disease or death. Patients who were lost to follow-up or died without evidence of disease were censored. Curves describing OS and EFS were generated by the Kaplan–Meier product limit method. The statistical significance of differences between the actuarial curves was tested by the log-rank test. Univariate and multivariate analysis was performed by Cox regression. Statistical analysis was performed in R (version 4.1.0; The R Foundation for Statistical Computing, Vienna, Austria) within R Studio (9, 10).

## Results

### Patient characteristics and treatment modalities

In total, 303 patients were included in this study. Patient characteristics and treatment modality are described in **Table 1**. The median age was 49 years (range, 1–91 years). Eighty-nine patients (29.4%) were female, and 214 (70.6%) were male. Most of the patients were diagnosed at an advanced stage, with 86 patients (28.4%) of T3 stage and 184 (60.8%) of T4 stage. Squamous cell carcinoma was the most common histologic type, followed by mesenchymal malignant tumors (arising from soft tissue, bone, cartilage, and lymphatic system), olfactory neuroblastoma, adenoid cystic carcinoma, malignant melanoma, adenocarcinoma, undifferentiated carcinoma, and other malignancies. Of the 261 patients treated by surgery, 194 patients (64.0%) received endoscopic surgery. More than half of the patients (53.8%) received post-operative adjuvant therapy, while only 39 (12.9%) received neoadjuvant chemotherapy. The recurrence rate was 21.8% in our institution.

**Table 1:** Patient characteristics of sinonasal malignancy.

Characteristic		Value
*n*		303
Age (median, range)		49 (1.91)
Sex = female (%)		89 (29.4)
T stage (%)	T1	11 (3.6)
	T2	22 (7.3)
	T3	86 (28.4)
	T4a	122 (40.3)
	T4b	62 (20.5)
N stage (%)	N0	279 (92.1)
	N1	3 (1.0)
	N2a	2 (0.6)
	N2b	8 (2.6)
	N2c	10 (3.3)
	N3b	1 (0.3)
Pathology (%)	Squamous cell carcinoma	81 (26.7)
	Mesenchymal malignant tumor	59 (19.5)
	Olfactory neuroblastoma	53 (17.5)
	Adenoid cystic carcinoma	32 (10.6)
	Malignant melanoma	28 (9.2)
	Adenocarcinoma	16 (5.3)
	Sinonasal undifferentiated carcinoma	10 (3.3)
	Not otherwise specific	24 (7.9)
Neoadjuvant chemotherapy (%)	Yes	39 (12.9)
Neoadjuvant efficacy evaluation (%)	CR	3 (7.7)
	PR	21 (53.8)
	SD	11 (28.2)
	PD	4 (10.3)
Treated by surgery (%)	Yes	261 (86.1)
Surgical approach (%)	Endoscopic approach	194 (74.3)
	Open approach	36 (13.8)
	Endoscopic with auxiliary incision	31 (11.9)
Surgical margin (%)	R0 resection	126 (48.3)
	R1 resection	108 (41.4)
	Tumor-debulking resection	27 (10.3)
History of radiation therapy (%)	Yes	19 (6.3)
Intraoperative 3D navigation (%)	Yes	29 (9.6)
Skull base reconstruction (%)	Yes	36 (11.9)
Adjuvant therapy (%)	Yes	163 (53.8)
Recurrence (%)	Yes	84 (27.7)
Locoregional recurrence (%)	Yes	64 (21.1)
Distant metastasis (%)	Yes	20 (6.6)

CR, complete remission; PR, partial remission; SD, stable disease; PD, progression of disease; R0, microscopically margin-negative resection; R1, macroscopic complete resection; Tumor-debulking resection, patients received tumor-debulking resection followed by radical radiotherapy.

### Oncologic results of SNM patients and different histologic subsets

**Figure 1:**
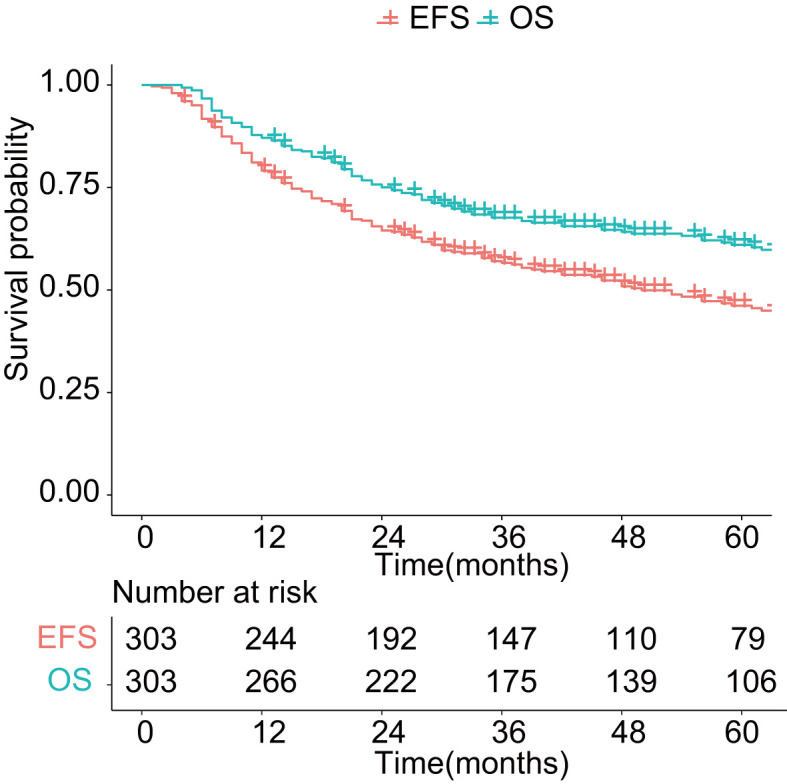
Overall survival and event-free survival in patients with sinonasal malignancies. OS: overall survival; EFS: event-free survival.

**Figure 2:**
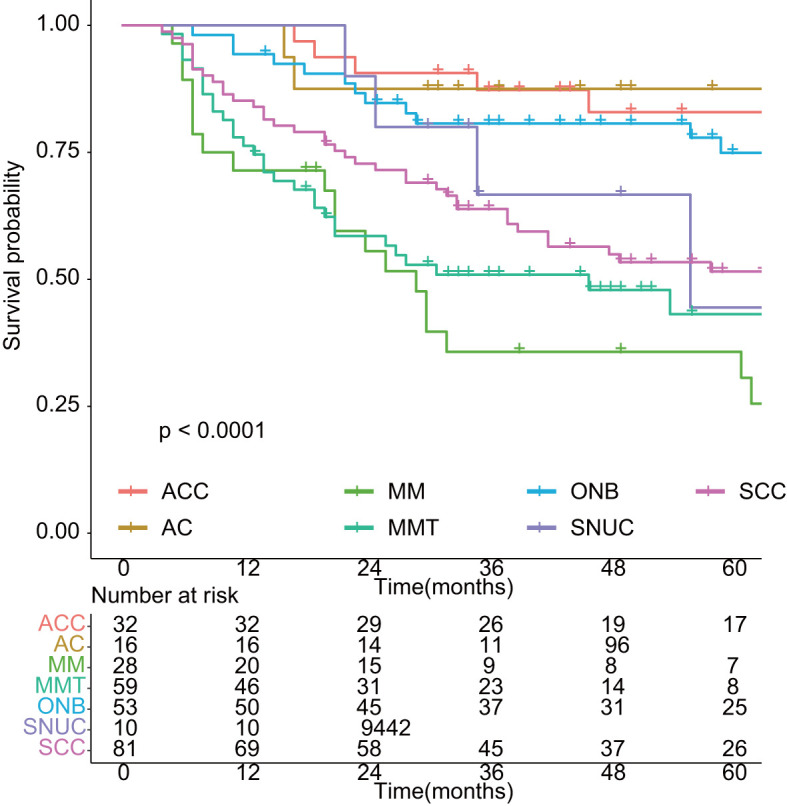
Overall survival in different histology of sinonasal malignancies. ACC: adenoid cystic carcinoma; AC: adenocarcinoma; MM: malignant melanoma; MMT: mesenchymal malignant tumor; ONB: olfactory neuroblastoma; SNUC: sinonasal undifferentiated carcinoma; SCC: squamous cell carcinoma.

As shown in **Figure 1**, with a median follow-up of 42 months (range, 4–201 months), the 3-year OS and EFS were 67.6% (95% CI: 62.5%–73.2%) and 56.6% (95% CI: 51.2%–62.6%), respectively. The 5-year OS and EFS were 61.0% (95% CI: 55.4%–67.1%) and 46.2% (95% CI: 40.4%–52.7%), respectively. The 10-year survival figures were 46.4% (95% CI: 39.2%–55.0%) and 31.3% (95% CI: 24.4%–40.1%), respectively. The 5-year OS differed significantly among different histologic types (*p* < 0.01, **Figure 2**). Malignant melanoma carried the worst prognosis at 5 years (35.7%, 95% CI: 21.3%–60.0%), followed by mesenchymal malignant tumor (43.1%, 95% CI: 30.4%–61.2%), sinonasal undifferentiated carcinoma (44.4%, 95% CI: 21.1%–65.5%), squamous cell carcinoma (51.5%, 95% CI: 41.2%–64.4%), olfactory neuroblastoma (74.9%, 95% CI: 63.3%–88.7%), and adenoid cystic carcinoma (79.1%, 95% CI: 70.1%–98.0%). Patients with adenocarcinoma had the best prognosis (87.5%, 95% CI: 72.7%–100%).

### Oncologic results of distinct treatment modalities

**Figure 3:**
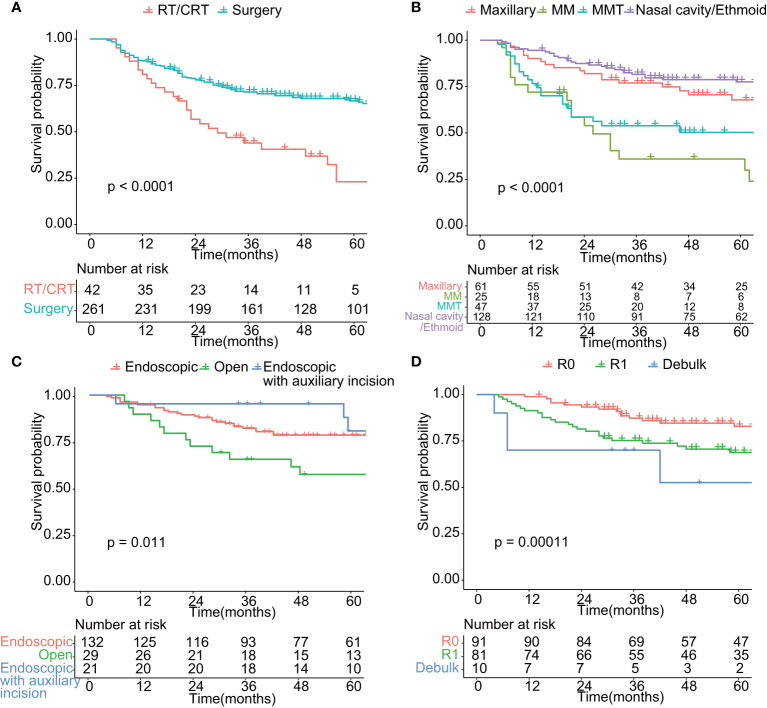
Comparison of overall survival in patients treated by different modality. **(A)** Comparison of overall survival of patients treated by surgery or radiotherapy/chemoradiotherapy (*p* < 0.01). **(B)** Comparison of overall survival of patients with different tumor site (*p* < 0.01). Maxillary: tumor located in maxillary sinus; MM: malignant melanoma; MMT: mesenchymal malignant tumor; Nasal cavity/Ethmoid: tumor located in nasal cavity or Ethmoid sinus. **(C)** Comparison of overall survival of patients undergoing surgery of different surgical approach (*p* < 0.05). Endoscopic: endoscopic approach; Open: Open approach; Endoscopic with auxiliary incision: endoscopic approach with auxiliary incision such as Caldwell-Luc incision and lateral rhinotomy. **(D)** Comparison of overall survival of patients undergoing surgery of different surgical purpose (*p* < 0.01). R0: Microscopically margin-negative resection (R0 resection); R1: Macroscopic complete resection (R1 resection); Debulk: Tumor-debulking resection. **(C, D)** included tumors of epithelial origin (*n* = 182).

To avoid possible confounding factors, we compared baseline characteristics of patients in early stage (T1–T2) and advanced stage (T3–T4). The distribution of age, gender, histology, surgical approaches, tumor origin, and surgical margin did not vary significantly between patients with early stage (T1–T2) and those with advanced stage (T3–T4) (**Supplementary Table 1**). As shown in **Figure 3**, the oncologic results differed from distinct treatment modalities. The 5-year OS was 66.6% (95% CI: 60.9%–72.9%) for patients who received surgery, better than those who only receive radiotherapy and chemotherapy with curative intent (5-year OS = 23.1%, 95% CI: 11.6%–45.8%, *p* < 0.01, **Figure 3A**). According to AJCC cancer staging manual, SNMs were divided into four categories by the origin and histology of the tumor, including maxillary sinus carcinoma, nasal cavity and ethmoid sinus carcinoma, head and neck sarcoma, and malignant melanoma. Tumors of epithelial origin, despite the primary location of the tumor, shows similar survival rates. Malignant melanoma and mesenchymal malignant tumor carried worse prognosis (**Figure 3B**). Due to limited cases and confounding impact on prognosis of malignant melanoma and mesenchymal malignant tumor, we only included tumors of epithelial origin in the following analysis. The prognosis of endoscopic surgery with auxiliary incision was superior to endoscopic surgery alone, while the open surgery shows the worst (*p* < 0.05, **Figure 3C**). R0 resection significantly benefited OS, while patients who received debulk surgery carried the worst prognosis (*p* < 0.01, **Figure 3D**). Moreover, endoscopic surgery has significantly less intra-operative bleeding (mean = 276.1 ml, *p* < 0.001) and shorter hospital stay (mean = 9.4 days, *p* < 0.001) compared to the open approach (mean = 488 ml, mean = 10.8 days).

**Figure 4:**
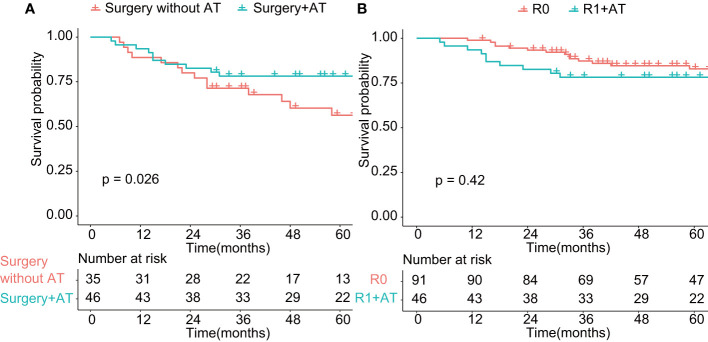
Comparison of overall survival in patients treated with adjuvant therapy (*p* < 0.05). AT: adjuvant therapy. **(A)** Comparison of overall survival of patients undergoing surgery with or without adjuvant therapy (*p* < 0.01). **(B)** Comparison of overall survival of patients undergoing R0 resection and patients undergoing R1 resection with adjuvant therapy (*p* > 0.05). R0: Microscopically margin-negative resection (R0 resection); R1 + AT: Macroscopic complete resection (R1 resection) with adjuvant therapy.

Adjuvant therapy had a documented role in the treatment of SNM patients who received R1 resection (*p* < 0.01, **Figure 4A**). We investigate the 5-year OS of patients who received R1 resection combined with adjuvant therapy and patients who underwent R0 resection and found no statistical significance of prognosis between the two groups (**Figure 4B**), which indicated the compensated role of adjuvant therapy in the case of positive margin. However, the role of neoadjuvant chemotherapy remains inconclusive for advanced-stage SNM (data not shown).

Univariate and multivariate analysis of risk factors for EFS were performed and reported in **Table 2**, with age, gender, tumor origin, AJCC T stage, radiotherapy history, sinonasal surgical history, neoadjuvant therapy, surgical approach, intraoperative 3D navigation, skull base reconstruction, margin, and adjuvant therapy as the covariates. Variables that were significant risk factors of lower EFS were older age (hazard ratio [HR] = 1.02, 95% confidence interval [CI] = 1–1.04; *p* = 0.02), R1 resection (HR = 1.99; 95% CI = 1.09–3.65, *p* = 0.02), sinonasal surgical history more than 3 months before diagnosis (HR = 2.77; 95% CI = 1.32–5.81, *p* = 0.007), and radiotherapy history (HR = 3; 95% CI = 1.37–6.55, *p* = 0.006). Univariate and multivariate analysis of risk factors for OS were performed and reported in **Table S2**. Variables that were significant risk factors of lower OS were older age (hazard ratio [HR] = 1.03, 95% confidence interval [CI] = 1.01–1.05; *p* = 0.02), R1 resection (HR = 1.87; 95% CI = 1.03–3.42, *p* = 0.041), and radiotherapy history (HR = 3.15; 95% CI = 1.45–6.83, *p* = 0.004). Skull base reconstruction significantly benefited OS of SNM patients (HR = 0.38; 95% CI = 0.01–0.90, *p* = 0.032). The probability of death doubled for patients over 40 years old, while the probability of recurrence increased by 58% for patients over 45 years old (**Figure S2**).

**Table 2:** Results of the univariable Cox regression analyses for event-free survival of SNM patients.

Characteristics		Univariate analysis			Multivariate analysis		
		HR	CI	*p*	HR	CI	*p*
Age		1.02	1–1.03	0.029	1.02	1–1.04	0.024
Gender	Female	ref					
	Male	0.64	0.36–1.15	0.133			
Origin	Maxillary	ref					
	Nasal cavity	0.71	0.42–1.19	0.198			
Stage	Early	ref					
	Advanced	1.72	0.78–3.8	0.176			
T stage	T1	ref			ref		
	T2	4.21	0.51–34.94	0.184	3.1	0.36–26.58	0.303
	T3	5.34	0.72–39.62	0.102	3.14	0.41–24	0.269
	T4a	3.65	0.48–27.54	0.209	2.5	0.32–19.34	0.381
	T4b	6.82	0.9–51.55	0.063	4.81	0.6–38.43	0.139
Surgical approach	Endoscopic	ref			ref		
	Endoscopic assisted surgery	2.24	1.26–3.97	0.006*	0.92	0.35–2.41	0.865
	Open	0.84	0.33–2.13	0.707	1.66	0.92–2.99	0.092
Surgical margin	R0	ref			ref		
	R1	2.55	1.48–4.38	0.001*	1.99	1.09–3.65	0.025*
History of radiation therapy	No	ref			ref		
	Yes	3.2	1.52–6.75	0.002*	3	1.37–6.55	0.006*
Intraoperative 3D Navigation	No	ref					
	Yes	1.08	0.43–2.72	0.866			
Skull base reconstruction	No	ref			ref		
	Yes	0.36	0.13–0.99	0.049*	0.42	0.14–1.23	0.115
Nasal surgical history	No	ref			ref		
	<3 Months	0.54	0.21–1.36	0.192	0.54	0.21–1.39	0.201
	≥3 Months	2.52	1.26–5.02	0.009*	2.77	1.32–5.81	0.007*
Neoadjuvant therapy	No	ref					
	Yes	1.22	0.58–2.57	0.6			
Adjuvant therapy	No	ref					
	Yes	0.75	0.45–1.26	0.277			

*p < 0.05.

R0, microscopically margin-negative resection; R1, macroscopic complete resection; Tumor-debulking resection, patients received tumor-debulking resection followed by radical radiotherapy.

## Discussion

Our current retrospective cohort of SNM patients attempted to provide comprehensive evidence for the application of surgery in the SNM multimodality treatment, with a special attention to the prominent role of R0 resection. The 5-year OS and EFS rates for SNM patients were 61.0% and 46.2%, respectively. The 5-year OS was poorest for malignant melanoma and best for adenocarcinoma. R0 resection carried the best prognosis while endoscopic surgery contributed to better prognosis of SNM patients as well. Our study is a large single-institution cohort with an extensive follow-up period. This is the largest single-institution cohort reported with a long-term follow-up. We specifically investigate risk factors of patients with recurrence. EFS is worse among patients with older age, R1 resection, sinonasal surgical history of more than 3 months before diagnosis, and radiotherapy history.

The oncologic outcomes of patients with SNM have been improving over the last 40 years (11, 12), which is probably attributed to newly developed surgical approaches, finer surgical techniques, and subsequent adjuvant treatment. Due to the long period of our cohort, we also compared the prognosis of patients in different periods. Patients treated after 2011 had better OS than patients treated before 2011 (*p* = 0.044, data not shown). This result is consistent with the moment when our institute started to promote multidiscipline treatment and update the surgical instrument. According to previous studies, the 5-year OS of SNM patients ranged from 69% to 82% (13, 14). In this study, the 5-year and 10-year OS were 59.6% and 47.6%, respectively. SNMs typically have an insidious onset, and most patients present with advanced disease (74.5% in our institute). Differences in the proportion of advanced-stage patients and various histologic types result in the discrepancy of OS between different medical centers. Tumor with advanced stage and worse biological behavior, including malignant melanoma and mesenchymal malignant tumor, accounted for a large proportion in our cohort, which may cause underestimation of OS. The tremendous pathologic diversity among SNMs complicates the development of a uniform and prognostically relevant staging system. Squamous cell carcinoma is the most common among all histologic subtypes of SNMs. A SEER Database Analysis reported that the 5-year OS was 58.6% for squamous cell carcinoma, which is comparable to our data (50.0%) (15). Almeida et al. suggested that malignant transformation of inverted papilloma does not have prognostic significance comparing *de novo* squamous cell carcinoma, which is entailed in our subgroup analysis (*p* = 0.34, **Figure S3**) (16). For olfactory neuroblastoma, the 5-year OS ranges from 51% to 97%, with a higher 5-year OS for patients treated endoscopically (17–20). The poor 5-year OS for malignant melanoma ranges from 20% to 55% in the literatures, similar to the poor prognosis in our cohort (21–23). Therefore, mixing all patients would conceal the actual oncologic outcome of different treatment modalities. Ferrari et al. suggested that the management of sinonasal cancer should be histology-driven (24). Based on the guidance of TNM staging and survival analysis, we include tumors that applied to TNM staging of nasal cavity, ethmoid sinus, and maxillary sinus cancer. However, our univariate Cox regression analysis suggested that the prognosis was not significant between early- and advanced-stage cancers (25). In multivariate analysis, only patients in the T4b stage present a significant worse prognosis. This suggested that SNMs’ TNM staging system may not be as clinically useful for predicting prognosis as the TNM system for other head and neck malignancies.

Unlike malignancies of trunks or extremities, excision of head and neck malignancies may find it hard to follow the oncologic surgery principle due to the complexity of anatomy. It is difficult for patients with advanced stage of tumors to receive an R0 resection and avoid disfigurement at the same time. Whether R1 resection is appropriate for acceptable oncologic outcome remains controversial. Patel et al. showed that, even in traditional open approaches, the margins of surgical resection are close to or positive in 31.6% of patients (5). Meanwhile, several scholars suggested that an en bloc resection is not superior to a piecemeal resection under the premise of negative surgical margin (6, 26). Our data show that a clear margin is critical in transnasal skull base surgery. In addition, the 5-year OS of patients who received R1 resection combined with adjuvant therapy did not yield worse prognostic difference compared to the patients with R0 resection. To the best of our knowledge, this is the first cohort to report a comparable oncologic outcome from patients who received R1 resection followed by adjuvant therapy. However, R0 resection showed a trend towards better prognosis. Furthermore, in the multivariate analysis, R0 resection conferred a better prognosis when compared to R1 resection, with a hazards ratio of 0.37 (*p* < 0.001). Moreover, debulk surgery should not be selected except for salvage intention only. A recent meta-analysis suggested that for more invasive and aggressive malignancies, salvage surgery following adjuvant therapy could provide the best opportunity for disease control (27). In our study, several patients were initially misdiagnosed as having nasal polyps and received R1 sinonasal surgery before transferring to our center. They were later diagnosed as having SNM and received R0 resection. We divided them into two categories according to the interval between two surgeries. Multivariate analysis suggested that having a salvage surgery more than 3 months after the first surgery increases the incidence of recurrence. Therefore, we should put emphasis upon the persistence to the oncologic surgery principle and the achievement of safety margin. The R0 surgery should not be abandoned because of the supplementary comprehensive treatment modalities.

Endoscopic approaches to the skull base and sinonasal regions offer several advantages. Apart from clear visualization of the nasal cavity and the tumor margin, endoscopic approaches avoid cosmetic deformities, brain retraction for tumor access, and resection. In the current analysis, endoscopic surgery carried better prognosis than the traditional open approach. In addition, the endoscopic surgery group has less bleeding during operation and shorter hospital stay. Similarly, Hanna et al. retrospectively reviewed 120 SNM patients who underwent endoscopic surgery with or without craniotomy and showed that margin status, disease recurrence, survival and post-operative complication rate did not differ significantly between the two groups (28, 29). A meta-analysis encouraged an endoscopic approach with a 5-year OS of 72.3% (25). For cases appropriate for R0/R1 resection endoscopically, endoscopic resection of SNM could lead to oncologic outcomes comparable to open surgery. However, the practice and cumulative experience of endoscopic surgical skill is necessary to reach a satisfying outcome. As for the indication of open surgery, Bossi et al. summarized that cranioendoscopic approach should be applied whenever tumor involves the dura laterally over the orbital roof, the brain tissue, or with a diffuse involvement of the frontal sinus, while transfacial approach should be applied whenever tumor extends to the maxillary sinus (with the exception of its medial wall), the nasal fossa bony floor, premaxillary and/or orbital soft tissues, lacrimal pathway, and/or infratemporal fossa (30). Nicolai et al. extended the indication of endoscopic surgery to include patients with skull base invasion and “focal” dural infiltration (14). Similarly, in the univariate analysis, we found that for skull base malignancies, dural excision followed by skull base reconstruction instead of ablation reduces the risk of recurrence (**Table 2**). Although this conclusion is not significant in multivariate analysis, dural excision followed by skull base reconstruction *via* minimally invasive endoscopic surgery enables R0 resection of tumors with limited dural invasion. Davide Mattavelli et al. suggested that for nasal-ethmoidal tumors with brain invasion, transnasal craniectomy and subpial dissection can provide satisfactory survival (31). Currently, differences in endoscopic equipment, surgical experience, and technique exist among institutions. No consensus is reached on the choice of surgical approach for SNM. Kılıç et al. performed propensity score-matched analysis of 652 patients and found that endoscopic surgery is an effective alternative to open surgery, even after accounting for confounding factors such as tumor size, tumor location, and TNM stage (32). Further studies on developing a commonly approved surgical classification system based in the tumor extent and the oncologic surgery principle are needed.

Alongside developments in surgery, there have also been improvements in radiotherapy and chemotherapy. Employment of systemic therapy for locally advanced disease could result in better outcomes and optimize the therapeutic armamentarium (33). Post-operative adjuvant therapy has a well-established role in the treatment of most craniofacial malignancies (34–37). The National Comprehensive Cancer Network suggested that adjuvant therapy should be applied except for T1 stage patients (38). In this study, patients received surgery and adjuvant therapy had better oncologic outcomes, which is consistent with the early studies (4, 39). However, toxicity in oncologic radiotherapy still needs attention. In our study, most patients lived in areas with high prevalence of nasopharyngeal cancer. According to multivariate analysis, patients who have a history of radiotherapy for treating nasopharyngeal carcinoma have a higher risk of SNM recurrence and worse prognosis. Given that radiotherapy may lead to several local and site-specific complications in the craniofacial region affecting the skin of the head, the eyes, and the brain, patients who achieved R0 resection could be exempted from post-operative adjuvant therapy. Due to the inadequate number of patients included, we did not find a significant difference between patients with and those without neoadjuvant therapy. Nevertheless, previous studies suggested that neoadjuvant chemotherapy can be advocated in the treatment of locoregionally advanced squamous cell carcinoma, sinonasal undifferentiated carcinoma, olfactory neuroblastoma, and craniofacial sarcoma (33, 40–43). Similarly, we encounter 24 individuals who partially or completely respond to neoadjuvant therapy (data not shown). The reduction of tumor size may allow patients to avoid open surgery and to increase the possibility of achieving a clear margin. In addition, neoadjuvant therapy could act as an indicator for the response to definitive concurrent chemoradiotherapy. For patients with advanced-stage malignancies who do not achieve a favorable response to neoadjuvant chemotherapy, Amit et al. suggested that surgery when feasible could provide a better chance of disease control and improved survival (41).

Several limitations exist in this study. Firstly, this is a retrospective cohort study that is prone to recall bias or misclassification bias. Secondly, due to the rarity of SNM, most of the patients treated in the early 2000s were lost to follow-up and were excluded from the study, and further follow-up is required to evaluate the prognosis of SNM patients of different histologic types. Thirdly, the heterogeneous cohort with mixed treatment regimens and unavailability of detailed schemes for adjuvant therapy might cause bias to this study. Finally, most of the patients did not report post-operation complications, resulting in the lack of data for the comparison of endoscopic surgery and open surgery.

## Conclusion

To the best of our knowledge, this is the largest cohort reported to date of SNM patients undergoing multimodality treatment in a single institution over an 18-year period. We found that curative-intent surgery has an irreplaceable role while the oncologic outcome of an endoscopic approach carried better prognosis than an open approach. Adjuvant therapy is necessary for all patients with R1 resection. The role of neoadjuvant chemotherapy remains inconclusive for advanced-stage SNM. A larger and histologic specific cohort is warranted on a multi-center basis in order to validate the current analysis.

## Data availability statement

The raw data supporting the conclusions of this article will be made available by the authors, without undue reservation.

## Ethics statement

The studies involving human participants were reviewed and approved by institutional review board of the first affiliated hospital of Sun Yat-sen University. The patients/participants' informed consent were waived.

## Author contributions

(I) Conception and design: Y-HW and W-PW. (II) Administrative support: Y-HW and W-PW. (III) Provision of study materials or patients: Y-HW and W-PW. (IV) Collection and assembly of data: M-YC, Y-HW, XW, YW, LC, Z-XH, TL, N-ZZ, and JL. (V) Data analysis and interpretation: M-YC, Y-HW, XW, and YW. (VI) Manuscript writing: All authors. (VII) Final approval of manuscript: All authors.

## Funding

This work was supported by the National Natural Science Foundation of China (NSFC) grants (81900918, 82020108009, and 81870696), Guangdong Natural Science Foundation of China grants (2018B030312008), Guangdong Research Program of Key Fields in Province (2020B1111300003), and the Key-Area Research and Development of Guangdong Province (2020B1111190001).

## Conflict of interest

All authors have completed the ICMJE uniform disclosure form. Y-HW reports that he is funded by the National Natural Science Foundation of China (NSFC) grants (81900918). W-PW reported that he was funded by the National Natural Science Foundation of China (NSFC) grants (82020108009 and 81870696), Guangdong Natural Science Foundation of China grants (2018B030312008), Guangdong Research Program of Key Fields in Province (2020B1111300003), and the Key-Area Research and Development of Guangdong Province (2020B1111190001).

The remaining authors declare that the research was conducted in the absence of any commercial or financial relationships that could be construed as a potential conflict of interest.

## Publisher’s note

All claims expressed in this article are solely those of the authors and do not necessarily represent those of their affiliated organizations, or those of the publisher, the editors and the reviewers. Any product that may be evaluated in this article, or claim that may be made by its manufacturer, is not guaranteed or endorsed by the publisher.
