# Supplementary figures and images for: Oncologic outcome of multimodality treatment for sinonasal malignancies: An 18-year experience
Source: Front Oncol. 2022 Sep 5;12:958142. doi: 10.3389/fonc.2022.958142 (PMC9484525; doi:10.3389/fonc.2022.958142)

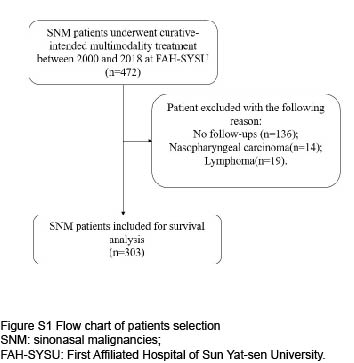

Supplement: Supplementary file 1 [file Image_1.jpeg]

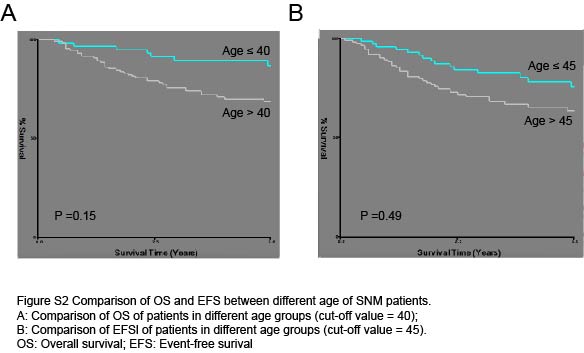

Supplement: Supplementary file 2 [file Image_2.jpeg]

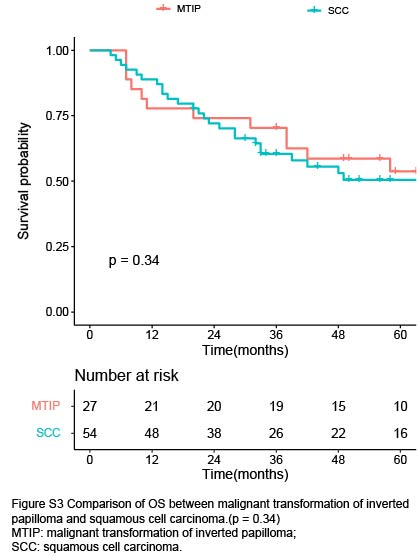

Supplement: Supplementary file 3 [file Image_3.jpeg]
