# Supplementary material for: Oncologic outcome of multimodality treatment for sinonasal malignancies: An 18-year experience
Source: Front Oncol. 2022 Sep 5;12:958142. doi: 10.3389/fonc.2022.958142 (PMC9484525; doi:10.3389/fonc.2022.958142)
Supplement: Supplementary file 4 [file Table_1.docx]

**Supplementary table 1 Comparison of baseline characteristics of SNM patients with early or advanced stage**

|  | level | Earlys tage | Advanced stage | p |
| --- | --- | --- | --- | --- |
| n |  | 28 | 161 |  |
| Gender (%) | Male | 17 (60.7) | 117 (72.7) | 0.289 |
|  | Female | 11 (39.3) | 44 (27.3) |  |
| Age (mean (SD)) |  | 48.75 (14.02) | 48.13 (17.00) | 0.856 |
| Pathology (%) | Adenoid cystic carcinoma | 5 (17.9) | 25 (15.5) | 0.011 |
|  | Adenoma | 7 (25.0) | 9 (5.6) |  |
|  | Olfactory blastoma | 3 (10.7) | 43 (26.7) |  |
|  | Other | 2 (7.1) | 20 (12.4) |  |
|  | Sinonasal undifferentiated carcinoma | 0 (0.0) | 6 (3.7) |  |
|  | Squamous cell carcinoma | 11 (39.3) | 58 (36.0) |  |
| Origin (%) | Maxillary | 10 (35.7) | 51 (31.7) | 0.839 |
|  | Nasal cavity | 18 (64.3) | 110 (68.3) |  |
| Surgical approach (%) | Endoscopic | 23 (82.1) | 114 (70.8) | 0.315 |
|  | Open | 4 (14.3) | 26 (16.1) |  |
|  | Endoscopic with auxiliary incision | 1 (3.6) | 21 (13.0) |  |
| Margin (%) | R0 | 16 (57.1) | 75 (46.6) | 0.408 |
|  | R1 | 12 (42.9) | 86 (53.4) |  |
| Neoadjuvant therapy (%) | no | 27 (96.4) | 137 (85.1) | 0.183 |
|  | yes | 1 (3.6) | 24 (14.9) |  |
