# Supplementary material for: Oncologic outcome of multimodality treatment for sinonasal malignancies: An 18-year experience
Source: Front Oncol. 2022 Sep 5;12:958142. doi: 10.3389/fonc.2022.958142 (PMC9484525; doi:10.3389/fonc.2022.958142)
Supplement: Supplementary file 5 [file Table_2.docx]

Table S2 Results of the univariable Cox regression analyses for Overall survival of SNM patients

| **Characteristics** |  |  | **Univariate analysis** |  |  | **Multivariate analysis** |  |
| --- | --- | --- | --- | --- | --- | --- | --- |
|  |  | **HR** | **CI** | **P** | **HR** | **CI** | **P** |
| Age |  | 1.02 | 1.01 - 1.04 | 0.007* | 1.03 | 1.01 - 1.05 | 0.002 |
| Gender | Female | ref |  |  |  |  |  |
|  | Male | 0.64 | 0.41 - 1.32 | 0.303 |  |  |  |
| Origin | Maxillary | ref |  |  |  |  |  |
|  | Nasal cavity | 0.73 | 0.43 - 1.22 | 0.227 |  |  |  |
| Stage | Early | ref |  |  |  |  |  |
|  | Advanced | 1.66 | 0.75 - 3.65 | 0.209 |  |  |  |
| T Stage | T1 | ref |  |  | ref |  |  |
|  | T2 | 3.46 | 0.42 - 28.81 | 0.251 | 1.85 | 0.21 - 16.14 | 0.578 |
|  | T3 | 4.21 | 0.57 - 31.31 | 0.160 | 2.35 | 0.31 - 17.91 | 0.411 |
|  | T4a | 3.31 | 0.44 - 24.98 | 0.246 | 2.21 | 0.29 - 17.07 | 0.447 |
|  | T4b | 6 | 0.79 - 45.25 | 0.082 | 5.23 | 0.66 - 41.65 | 0.118 |
| Surgical Approach | Endoscopic | ref |  |  | ref |  |  |
|  | Endoscopic assisted surgery | 0.75 | 0.30 - 1.92 | 0.553 | 0.75 | 0.29 - 1.95 | 0.552 |
|  | Open | 2.1 | 1.18 - 3.73 | 0.011* | 1.76 | 0.96 - 3.20 | 0.066 |
| Surgical margin | R0 | ref |  |  | ref |  |  |
|  | R1 | 2.75 | 1.56 - 4.83 | <0.001* | 1.87 | 1.03 - 3.42 | 0.041* |
| Post-radiation History | No | ref |  |  | ref |  |  |
|  | Yes | 3.07 | 1.45 - 6.47 | 0.003* | 3.15 | 1.45 - 6.83 | 0.004* |
| Intraoperative 3D Navigation | No | ref |  |  |  |  |  |
|  | Yes | 1.39 | 0.55 - 3.53 | 0.486 |  |  |  |
| Skull Base Reconstruction | No | ref |  |  | ref |  |  |
|  | Yes | 0.38 | 0.14 - 1.04 | 0.059 | 0.31 | 0.10 - 0.90 | 0.032* |
| Nasal Surgical history | No | ref |  |  |  |  |  |
|  | <3 Months | 0.53 | 0.21 - 1.34 | 0.18 |  |  |  |
|  | ≥3 Months | 1.71 | 0.86 - 3.38 | 0.126 |  |  |  |
| Neoadjuvant Therapy | No | ref |  |  |  |  |  |
|  | Yes | 1.34 | 0.63 - 2.84 | 0.445 |  |  |  |
| Adjuvant Therapy | No | ref |  |  |  |  |  |
|  | Yes | 0.77 | 0.46 - 1.28 | 0.312 |  |  |  |

*p<0.05

R0: microscopically margin-negative resection; R1: Macroscopic complete resection; Tumor-debulking resection: patients received tumor-debulking resection followed by radical radiotherapy.
